# Characterizing brain structures associated with subjective cognitive decline and mild cognitive impairment: a population‐based study

**DOI:** 10.1002/alz.088245

**Published:** 2025-01-09

**Authors:** Ziwei Chen, Qianqian Xie, Jiafeng Wang, Huisi Zhang, Chunyan Li, Lin Cong, yongxiang wang, Lin Song, Yifeng Du, Chengxuan Qiu

**Affiliations:** ^1^ Shandong Provincial Hospital Affiliated to Shandong First Medical University, Jinan, Shandong China; ^2^ Shandong Provincial Hospital Affiliated to Shandong First Medical University, Jinan China; ^3^ Shandong Provincial Clinical Research Center for Neurological Diseases, Jinan China; ^4^ Shandong Provincial Hospital, Shandong University, Jinan China; ^5^ Aging Research Center and Center for Alzheimer Research, Department of Neurobiology, Care Sciences and Society, Karolinska Institutet‐Stockholm University, Stockholm Sweden; ^6^ Shandong Provincial Hospital affiliated to Shandong First Medical University, Jinan China; ^7^ Shandong Provincial Hospital Affiliated to Shandong University, Jinan China; ^8^ Shandong Provincial Clinical Research Center for Neurological Diseases, Jinan, Shandong, P.R. China., Jinan China; ^9^ Department of Neurology, Shandong Provincial Hospital, Shandong University, Jinan, Shandong, P.R. China., Jinan China; ^10^ Aging Research Center, Karolinska Institutet and Stockholm University, Stockholm Sweden; ^11^ Aging Research Center and Center for Alzheimer Research, Karolinska Institutet‐Stockholm University, Stockholm Sweden; ^12^ Department of Neurology, Shandong Provincial Hospital affiliated to Shandong First Medical University, Jinan, Shandong, P.R. China., Jinan China

## Abstract

**Background:**

The structural brain alterations for subjective cognitive decline (SCD) and mild cognitive impairment (MCI) are poorly defined. We sought to characterize grey matter volume (GMV) and cortical thickness associated with SCD and MCI among rural‐dwelling older adults in China.

**Method:**

This population‐based cross‐sectional study included 1072 dementia‐free participants derived from the brain MRI substudy of MIND‐China in 2018‐2020. We defined MCI following the Petersen’s criteria, and SCD as the self‐reported AD8 questionnaire score ≥2. Data were analyzed using voxel‐based morphometry (VBM), surface‐based morphometry analysis (SBM), and logistic regression models.

**Result:**

SCD was defined in 243 individuals and MCI in 246 individuals. VBM analyses revealed a significant reduction in GMV among people with MCI compared to normal controls (NC) in several brain regions, such as bilateral temporal lobes and bilateral precuneus (P<0.05). There were no significant differences in GMV between SCD and NC (P<0.05). The ROI‐wise SBM analyses revealed that SCD was significantly associated with cortical thinning in the right paracentral sulcus, left caudal middle frontal gyrus, and entorhinal cortex (P<0.05) and that MCI was significantly associated with cortical atrophy in the left caudal middle frontal gyrus, temporal lobe, frontal lobe, and parietal lobe (P<0.05).

**Conclusion:**

The structural brain altercations are characterized by reduced GMV mainly in the bilateral temporal lobe and bilateral precuneus, and thinner cortical thickness in the temporal lobe, frontal lobe, and parietal lobe for MCI, and by cortical thinning in the right paracentral sulcus, left caudal middle frontal gyrus, and entorhinal cortex for SCD.

